# Top-emitting quantum dots light-emitting devices employing microcontact printing with electricfield-independent emission

**DOI:** 10.1038/srep22530

**Published:** 2016-03-02

**Authors:** Shihao Liu, Wenbo Liu, Wenyu Ji, Jing Yu, Wei Zhang, Letian Zhang, Wenfa Xie

**Affiliations:** 1 State Key Laboratory on Integrated Optoelectronics, College of Electronic Science and Engineering, Jilin University, Changchun, 130012, People’s Republic of China; 2 Department of Physics, Quanzhou Normal College, Quanzhou 362000, People’s Republic of China; 3 Department of Physics, Jilin University, Changchun, 130012, People’s Republic of China

## Abstract

Recent breakthroughs in quantum dot light-emitting devices (QD-LEDs) show their promise in the development of next-generation displays. However, the QD-LED with conventional ITO-based bottom emission structure is difficult to realize the high aperture ratio, electricfield-independent emission and flexible full-color displays. Hence, we demonstrate top-emitting QD-LEDs with dry microcontact printing quantum dot films. The top-emitting structure is proved to be able to accelerate the excitons radiative transition rate, then contributing to stable electroluminescent efficiency with a very low roll-off, and preventing spectra from shifting and broadening with the electric field increases. The results suggest potential routes towards creating high aperture ratio, wide color gamut, color-stable and flexible QD-LED displays.

With the improvement of electronic technology, people’s desire for higher-performance displays which should have the properties of high quality fast-moving images, lightweight and low power consumption, becomes more desirable recently. The organic light-emitting devices (OLEDs) and quantum-dots light-emitting devices (QD-LEDs) would be probably promising ways to offer the satisfying new display technologies. Researches on the OLEDs could date back to the sandwich-like organic electroluminescent device fabricated by C. W. Tang and S. A. VanSlyke in 1987^1^. After several decades of development, the OLED technology becomes mature gradually and begins to come into our life lately^2^,^3^,^4^,^5^,^6^,^7^. Nevertheless, although electroluminescent (EL) QD-LEDs just grow up dramatically in recent years^8^,^9^,^10^, they have many better luminescent properties in comparison with the OLEDs, such as better color purity, longer lifetime and lower costs, which could be attributed to the corresponding QDs’ material characteristics, such as narrower emission spectra, better stability and cheaper raw materials^8^,^9^,^10^,^11^,^12^,^13^,^14^,^15^,^16^,^17^,^18^. Besides, the greatest advantage of the QDs for light-emitting applications is their size-controlled bandgap, due to different emitting colors could be obtained just through changing the size of the core^19^,^20^,^21^,^22^. Thus, the EL QD-LEDs becomes one of the research hotspots in optoelectronic field.

Based on QDs, excellent performance QD-LEDs that are catching up with the OLEDs in efficiencies and luminance have been reported by researchers^8^,^23^,^24^. Additionally, the full-color QD-LED-based displays, in which the corresponding red, green and blue element fabricated by dry pick-and-place transfer printing exhibits a maximum current efficiency of about 4.0, 0.5 and 0.04 cd A^−1^, respectively, are first fabricated successfully by Kim *et al*. proving the feasibility of QD-LEDs-based displays^25^. However, except Sun’s works^26^, almost all hitherto existing investigations on QD-LEDs are focused on the bottom-emission structure, of which emission light is emitting in the substrate direction. For the active matrix display based on bottom-emitting structure, its driving transistor sharing the substrate with the emission aperture would adversely limit the pixel aperture ratio, for example, the aperture ratio of 4-inch displays reported by Kim *et al*. is only 25%^25^. In addition, in most of QD-LEDs, when the bias voltages increase, the EL spectra could not remain unchanged easily due to the field-induced quantum confinement Stark effect (QCSE) that the varying electricfield would cause spontaneous spectral diffusion and then lead to ensemble inhomogeneous broadening^27^,^28^. Besides, the QCSE also have been identified to induce photoluminescence quenching of QDs, resulting in the high efficiency roll-off in the QD-LEDs^29^. Such electricfield-dependent emission spectra and high efficiency roll-off would severely impede the QD-LEDs from being practically applied to the high-performance displays.

Nevertheless, these disadvantages could be weakened by employing the top emitting structure, which have been demonstrated to be beneficial to device performances by studies on OLEDs^30^. In top-emitting device, the emission light passes through semitransparent top electrodes, enlarging the choices of the substrate materials and expanding the devices’ scope of application. Besides, the top-emitting structure allows the driving transistor to be placed under the light-emitting device, leading to better compatibility with the active matrix driving integrated circuits, larger pixel aperture ratio and higher resolution. Furthermore, due to the microcavity effect, the top-emitting structure also has a relatively positive effect on the optical and electrical properties, such as higher luminance, emission efficiency, color purity and lower roll-off for the light-emitting devices, which has also been studied by current researches on OLEDs^31^,^32^,^33^,^34^,^35^,^36^,^37^,^38^,^39^. Considering efficient charge injection, high light outcoupling and convenient experimental technical process, a thicker metal thin film is usually used as the lightproof bottom electrode, and a thinner one is for the semitransparent top electrode^40^. The metal thin film electrode always has high reflectivity so that the device system would form a resonator with strong microcavity effect^41^,^42^,^43^. Through introducing the electromagnetic boundary conditions, the microcavity effect would strongly alter the photon density of states^44^, which would obviously affect the optical properties of spontaneous emission in such a cavity, resulting in narrowed spectral linewidth, shortened spontaneous emission lifetime and enhanced directional emission. Thus, the Stark broadening and redshift phenomena of the EL spectra and high roll-off for the QD-LEDs would potentially be weakened by employing the top-emitting structure. Consequently, employing this MCP technology and thermal evaporation, TEQD-LEDs based on the core/shell CdSSe/ZnS are fabricated successfully and their optical and electrical properties are investigated.

The fabrication process of the TEQD-LEDs is shown in the Fig. 1. In the top-emitting devices, thick metal film used as the bottom electrode is not perfectly compatible with the wet process as it would increase the possibility of short circuit to a certain extent. Additionally, the wet process is difficult to pattern the individual RGB QDs in the full-color display panel and easily generates the cross-contamination of the RGB pixels. Thus, QD-LEDs with organic small molecular CTLs which could be fabricated by thermal evaporation and MCP, are the choice for TEQD-LEDs in this paper.

The standard MCP technique is accomplished by virtue of the elastomer stamp commonly formed by polydimethylsiloxane (PDMS)^45^,^46^. As seen in Fig. 1, the QDs solution is spin-coated on the PDMS elastomer stamp firstly, and then the PDMS stamp is transferred on the substrate which has been coated by the bottom electrode and CTLs. Through the conformal contact of the elastomeric stamp and the substrate, the QDs on the PDMS stamp would be moved on to the bottom CTLs. Nevertheless, the PDMS elastomer stamp is readily swelled by nonpolar solvents used to disperse the QDs, such as toluene, chloroform, cyclohexane and so on. Therefore, for the sake of preventing the swelling, a Parylene-C or SU-8 photoresist layer is proposed as a protective layer^45^,^46^. However, the chemical vapor deposition which is essential for the fabrication of Parylene-C makes the fabrication process more complicated and expensive. Additionally, after the UV radiation treatment, the SU-8 would become rigid, resulting in that the PDMS stamp loses its excellent mechanical property. Hence, in this paper, we propose a new approach with low cost and simple process in which only several micrometers oxidation layer formed by UV/ozone treatment is used as the protective layer for PDMS.

To avoid being swelled and increase chemical compatibility with toluene solutions of QDs, PDMS has been treated by ultraviolet light (UV) and ozone before inked by the QDs solution. The oxidizing treatment would form a thin glass-like SiO_x_ protective layer with just several micrometers and make the intact molecules retain the methyl side-group so that the mechanical property of PDMS stamp would not be affected dramatically^47^,^48^,^49^. Additionally, as shown in Fig. 2, the root mean square (RMS) roughness for the PDMS stamp before and after the UV/ozone treatment is 9.28 and 3.57 Å, respectively, indicating that the oxidizing treatment doesn’t have obvious effect on the surface topography of PDMS stamp. Furthermore, the phenomenon that the toluene wets the treated PDMS more efficiently as indicated by the decrease in the contact angle from 38.1° for toluene on untreated PDMS to 9.9° for toluene on treated PDMS is also shown in Fig. 2. The solvent-compatible would probably lead to a more uniform QDs film during spin-coating^46^.

Employing MCP process based on UV/ozone-treated PDMS, red, green and blue TEQD-LEDs are fabricated successfully, and the properties of the red TEQD-LEDs (RTEQD-LED), of which structure, energy level schemes, fluorescence microscopy image and scanning electron microscope (SEM) image are shown in the Fig. 3 are detailedly discussed in this paper. The devices share the structure: Ag/MoO_3_/Di-[4-(N,N-ditolylamino)-phenyl]cyclohexane (TAPC)/4,4′,4″-tris(carbazol-9-yl)-triphenylamine (TCTA)/QDs layer/2,2′,2″-(1,3,5-Benzinetriyl)-tris(1-phenyl-1-H-benzimidazole) (TPBI)/4,7-Diphenyl-1,10-phenanthroline (Bphen)/8-Hydroxy-quinolinolato-lithium (Liq)/Ag; and QDs of the devices are the core/shell CdSSe/ZnS (photoluminescence (PL) quantum yields >40%) with the corresponding size. A 22 nm Ag thin film is chosen as the semitransparent cathode due to its efficient charge injection, high light outcoupling and convenient experimental technical process. In the resonator formed by the high reflecting anode and cathode, the influence of interference effects is even stronger, resulting in that the emission wavelength would be affected by the cavity length^50^,^51^,^52^. Thus, the thickness of organic electron transfer layers (ETLs) and hole transfer layers (HTLs) has been estimated precisely to ensure the corresponding resonant wavelength to accord with the EL emission wavelength of QDs. From the fluorescence microscope and SEM images, it can be estimated that the top-emitting devices possess a uniform QD film, indicating the feasibility of microcontact printing process. Additionally, in Fig. 3(d), it can be also found that the thickness of QD film in the top-emitting device is about 10 nm.

Firstly, the electrical properties of the RTEQD-LED are discussed and its current density-voltage-luminance and current efficiency (CE)-luminance-external quantum efficiency (EQE) characteristics are shown in Fig. 4. For comparison, the corresponding red bottom emitting ITO-based QD-LED (RBEQD-LED) and inverted hybrid organic-inorganic QD-LED (RIBEQD-LED) are also fabricated successfully, and their performances are also shown in Figs 4 and 5. Additionally, the RBEQD-LED has the same identical structure with the RTEQD-LED except for the electrodes, while the RIBEQD-LED for comparison adopts the structure: ITO/ZnO (45 nm)/CdSSe/ZnS QDs/CBP(45 nm)/MoO_3_(5 nm)/Al (100 nm), which has been demonstrated as an efficient structure in the reported papers^28^,^53^,^54^. The turn-on voltage for RTEQD-LED, RBEQD-LED and RIBEQD-LED is 3.4, 3.8 and 2.7 V, respectively. The low turn-on voltage indicates the efficient carrier injection which could lead to low operating voltage and power consumption. From Fig. 4(a), it can be seen that the current densities of QD-LEDs employing thermal evaporating organic CTLs are relatively lower than that of RIBEQD-LED, which could be attributed to the poorer carrier mobility of the organic electron transfer layers in comparison with the ZnO^55^,^56^,^57^. As a result of the better carrier transport property of the inorganic material, RIBEQD-LED attains its maximum luminance (20620 cd/m^2^) at fairly low bias voltage (7.5 V), while the RTEQD-LED and RBEQD-LED realizes its maximum luminance of 8659 cd/m^2^ at the 10 V and 1466 cd/m^2^ at the 11.5 V, respectively. However, just consider the luminance for the devices only with organic CTLs, the value of RTEQD-LED is nearly 6-fold higher than that of the RBEQD-LED. Due to the relative low luminance and poor carrier transport of organic CTLs, the maximum current efficiency of RBEQD-LED is only 0.81 cd/A and 0.34%, respectively, as shown in Fig. 4(b). However, although the RTEQD-LED has lower luminance, it possesses a higher efficiency than the RIBEQD-LED, as indicated by that the maximum current efficiency of the RTEQD-LED is 4.40 cd/A and 1.62%, while that of RIBEQD-LED is only 3.20 cd/A and 1.46%, respectively. Apparently, in comparison to RBEQD-LED, RTEQD-LED exhibits remarkable enhancement in EL luminance and efficiency, which could be attributed to the accelerating excitons radiative transition rate caused by the microcavity effect.

The existence of microcavity would strongly enhanced local electromagnetic field of the device, resulting in the changes of the photonic mode density^44^. The photonic mode density near the cavity mode would be enhanced, and others would be weakened. As the spontaneous radiative transition rate is proportional to the photonic mode density, the corresponding spontaneous radiative transition rate would increase, resulting in enhanced luminescence. It can be seen from Fig. 5(a) that the PL lifetime of red QDs reduces from 17.87 ns in RBEQD-LED to 13.87 ns in RTEQD-LED, make clear that the excitons radiative transition rate has been accelerated in RTEQD-LED. Additionally, the shorter lifetime of the excitons in RTEQD-LED would be beneficial to weaken the excitons quenching, and then lead to a lower EL efficiency roll-off. Fig. 4(b) shows that the current efficiency of BEQD-LED at its maximum luminance (1466 cd/m^2^) only reaches 40.7% of its maximum efficiency, while the corresponding proportion for IBEQD-LED is 63.0%. However, the EL efficiency TEQD-LED possesses more stability as its current efficiency at the maximum luminance (8659 cd/m^2^) could attain to 84.1% of its maximum.

Secondly, the RTEQD-LED also has obvious superiority on the color purity relative to the bottom-emitting ones. The normalized EL spectra of RTEQD-LED, RBEQD-LED and RIBEQD-LED at the luminance of 1000 cd/m^2^ are shown in the Fig. 5(b). In the Figure, only the purely intrinsic red emissions are seen in the obviously narrowed spectra of RTEQD-LED, while slight blue and red emissions which are considered to be emitted by the organic CTLs or their excimers^58^,^59^ could be found in the spectra of RBEQD-LED. Additionally, compared with the RBEQD-LED (46 nm) and RIBQD-LED (44 nm), because of the microcavity, the FWHM of the RTEQD-LED device is narrowed about 32%, revealing that the RTEQD-LED would offer better color saturation and larger color gamut for the full-color QDs display screens. The narrower emission for the RTEQD-LED should also be attributed to the microcavity effect. The light emitted in the microcavity is typically captured in the single, spatially well-defined mode of the field radiating from the microcavity. Thus, the emission with the corresponding wavelength would be enhanced strongly, while others would be weakened, leading to the narrower FWHM.

As the same reason above, unlike the bottom-emitting devices, the RTEQD-LED also exhibits relatively electricfield-independent EL emission spectra. Figure 6 shows the EL spectra of the RTEQD-LED under different bias voltages. As the bias voltage increases, the peak emission wavelength (λ_max_) for RTEQD-LED remains unchanged at the fixed wavelength of 600 nm, and the normalized EL spectra are absolutely identical. The CIE coordinates of RTEQD-LED just shift (−0.005, 0.000) from 4 V to 9 V in its operating voltage range, while those of RBEQD-LED and RIBEQD-LED are (−0.013, −0.004) from 6 V to 10 V and (−0.011, −0.003) from 4 V to 7 V, respectively, confirming that the EL spectra for RTEQD-LED possess considerable stability, which could be explained by the resonance interaction formed by the resonant cavity existing in RTEQD-LED. Hence, given all these points above, the top-emitting structure could be considered to be a useful method for fabricating efficient and purely monochromatic QD-LEDs employing organic CTLs. Furthermore, according to the theory of Fabry-Pérot resonator^41^, the resonance wavelength at 600 nm could also indicate that the thickness of QD film in RTEQD-LED is approximately 10 nm, according with the conclusion obtained from the cross-sectional SEM image.

And lastly, like the top-emitting OLEDs, the RTEQD-LED also shows a preferential forward-direction emission. The relative EL intensity of the spectra for RTEQD-LED at different viewing angles is shown in Fig. 7. It can be seen from the Fig. 7 that as the viewing angle increases from 0° to 60°, the peak wavelength of the spectra only blue-shift 2.5 nm close to the just noticeable difference that the human could discriminate^60^,^61^. Besides, a more forward directed emission relative to a Lambertian distribution could be also clearly observed in the RTEQD-LED. Such forward directed emission are usually considered to be adverse to wide-viewing angle visibility, but it could be used to realize the privacy-protection screen with forward-prior luminance and stable color hues for individual laptops and smartphones that demand more information security than convenience for co-working.

The superiorities of the top-emitting structure are also equally applicable to the green and blue QD-LEDs as the TEQD-LEDs based on blue or green emission QDs still exhibits significant improvements in luminance, EL efficiency and color purity as shown in Table 1. Additionally, for the reason that the emission light is emitting through top electrodes in the top-emitting structure, it is convenient to enhance the electrical and optical properties for QD-LEDs by employing suitable light-outcoupling layer, such like organic, dielectric layers or patterned microlens films^4^,^50^,^62^,^63^,^64^,^65^,^66^.

## Conculsion

In summary, efficient contact-printing monochromatic TEQD-LEDs employing top-emitting structure are demonstrated. Compared to the conventional QD-LEDs with bottom-emitting structure, there are significant improvements in efficiency, luminance and color purity for TEQD-LEDs. The device performances of the red TEQD-LED are discussed in detail, and it shows a maximum luminance of 8695 cd/m^2^ and a maximum current efficiency of 4.40 cd/A, which could be comparable to those of the ones with efficient hybrid CTLs. Besides, the red TEQD-LED also possesses extremely stable emission spectra under different bias voltages and a very low efficiency roll-off. Thus, the work would be beneficial to the development of efficient QD-LEDs displays with high aperture ratio, wide color gamut and stable color.

## Methods

### Device fabrication

The organic materials used in our experiments are purchased from Luminescence Technology Corporation. All devices are fabricated on glass substrates which are subjected to a routine cleaning process with rinsing in Decon 90, deionized water, drying in an oven, and finally treating in a plasma cleaner chamber. The electrode and small molecular organic CTLs are evaporated by thermal evaporation on the precleaned glass substrates under high vacuum (~5 × 10^−4^ Pa) with once breaking the vacuum for QDs deposition. A shadow mask is used to define the anode and cathode, and to make four 10 mm^2^ devices on each substrate. All the organic layers are thermally deposited in vacuum at a rate of 1–2 Å s^−1^ monitored *in situ* with a quartz oscillator. And before QDs deposition, anode and hole transport layers (HTLs) have been evaporated on the precleaned glass substrate.

### QD deposition

The PDMS elastomer stamps are made by mixing PDMS base and curing agent (Dow Corning Sylgard 184 silicone elastomer) with a mixing ratio of 10:1. The PDMS mixture is then pour onto a silicon master and curing at 60 °C for 1 h. After that, the cured PDMS is peeled from the silicon master and transferred onto the cleaned glass for the next step. Finally, for purpose of increasing chemical compatibility with the solution of QDs and avoiding being swelled, the PDMS receives a UV-ozone treatment for 3 h. The toluene solutions of core/shell CdSSe/ZnS purchased from NNCrystal are spin coated at 1200 rpm onto the treated PDMS. Subsequently, after annealing at 60 °C for 15 min in the atmosphere, the inked PDMS stamp is then brought into conformal contact with the HTLs substrate for 30 s, and then peeled away.

### Device characterization

The static contact angles are measured using a contact angle goniometer (JC2000D, Powereach Co., Shanghai, China). The fluorescence microscopy image is obtained by the inverted fluorescence microscope (Mshot M53, Micro-shot Technology Co. Ltd., Guangzhou, China). The cross-section SEM image of the top-emitting QD-LED device is characterized by Hitachi S4800 scanning electron microscope. Luminance-current-voltage characteristics of unpackaged devices are measured simultaneously using a Keithley 2400 Source Meter and a Minolta Luminance Meter LS-110 in air at room temperature. The spectra of the devices are recorded with an Ocean Optics Maya 2000-PRO spectrometer.

## Additional Information

**How to cite this article**: Liu, S. *et al*. Top-emitting quantum dots light-emitting devices employing microcontact printing with electricfield-independent emission. *Sci. Rep.* **6**, 22530; doi: 10.1038/srep22530 (2016).

## Figures and Tables

**Figure 1:**
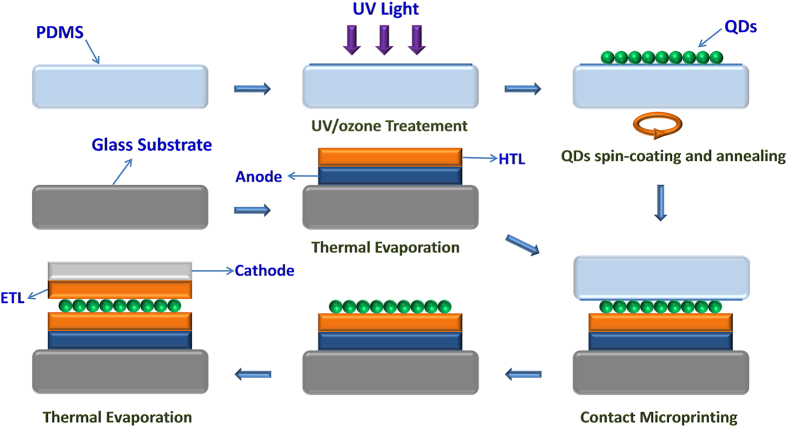
The fabrication process flow diagram of the TEQD-LEDs.

**Figure 2:**
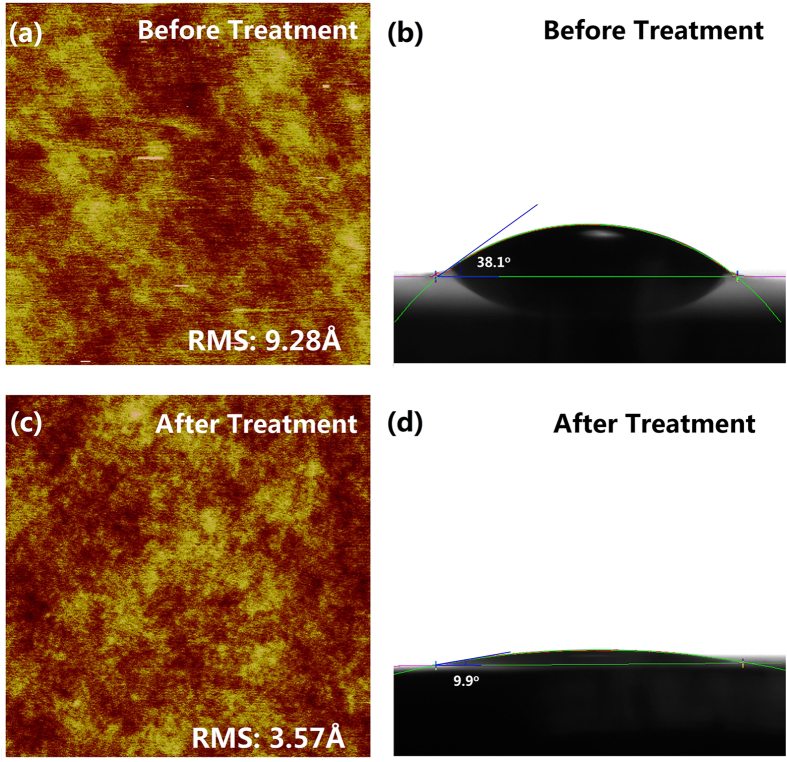
The (**a,c**) AFM topography (1 μm × 1 μm) and (**b,d**) surface toluene contact angle images for PDMS before and after the UV/ozone treatment.

**Figure 3:**
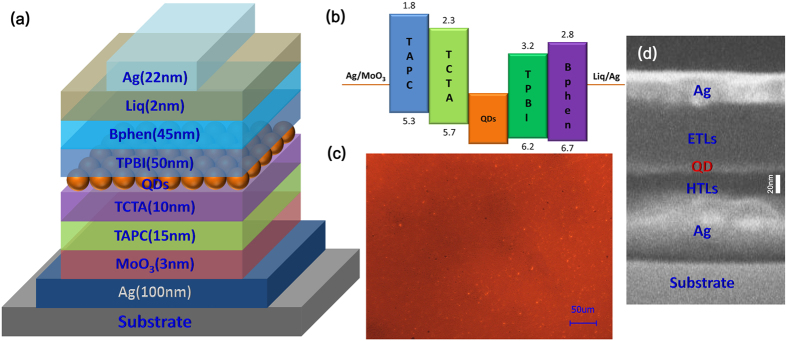
The (**a**) structure, (**b**) energy level schemes, (**c**) fluorescence microscopy image (green 510 nm excitation) and (**d**) cross-sectional SEM image of the RTEQD-LEDs.

**Figure 4:**
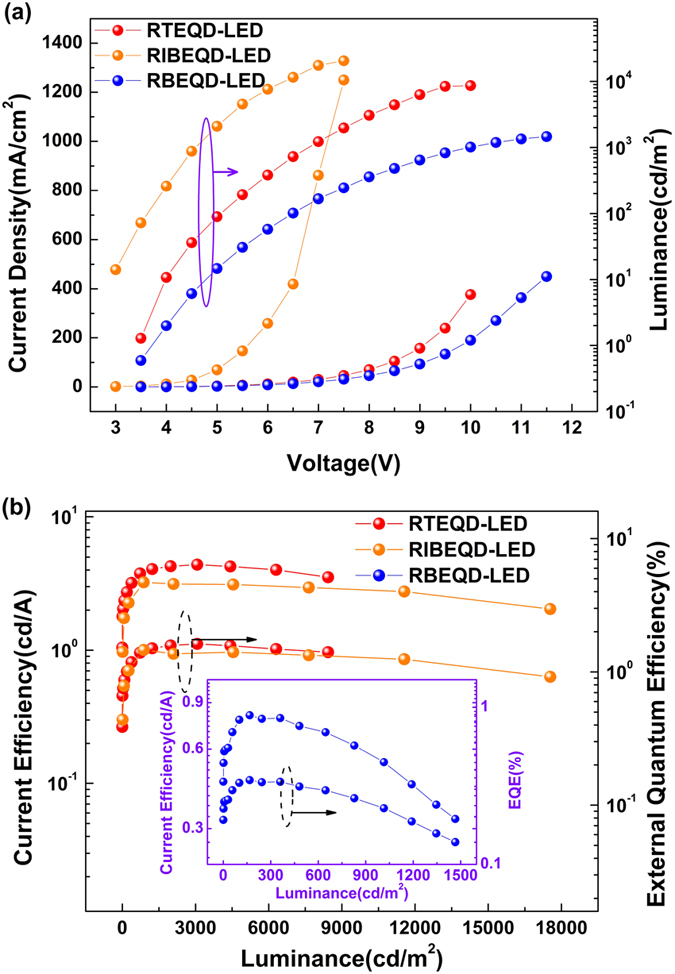
(**a**) Current density-voltage-luminance and (**b**) current efficiency-luminance-external quantum efficiency characteristics of RTEQD-LED, RBEQD-LED and RIBEQD-LED.

**Figure 5:**
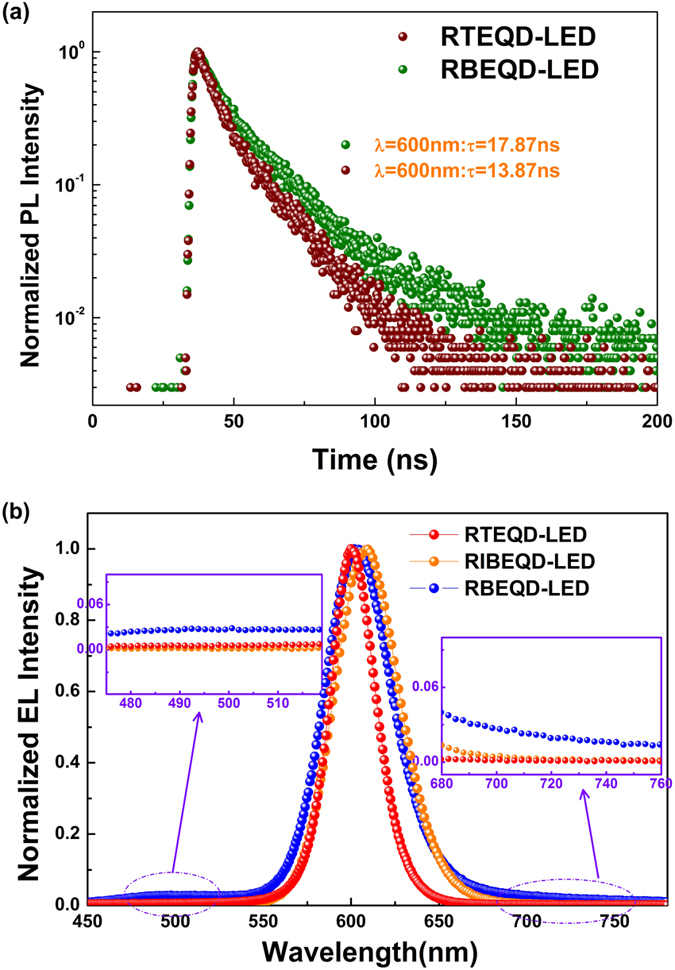
(**a**) The PL decay transients for QDs in RTEQD-LED and RBEQD-LED. (**b**) The normalized EL spectra of RTEQD-LED, RBEQD-LED and RIBEQD-LED at 1000 cd/m^2^.

**Figure 6:**
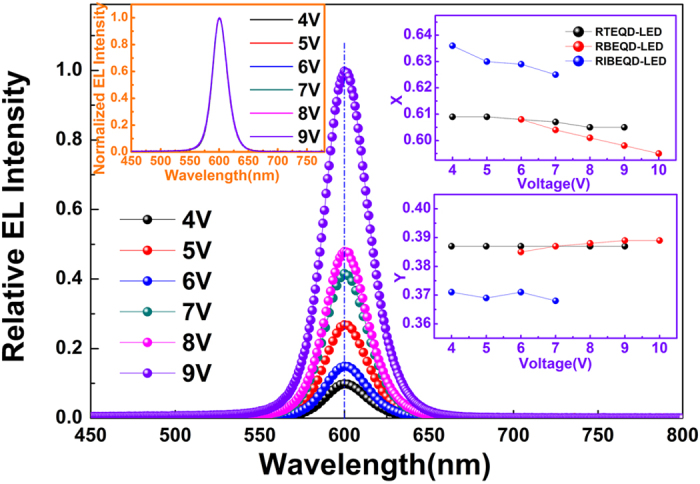
The relative EL spectra of the RTEQD-LED under different bias voltages. The insets are the normalized EL spectra for RTEQD-LED and CIE coordinates for RTEQD-LED, RBEQD-LED and RIBEQD-LED under their corresponding operating voltages.

**Figure 7:**
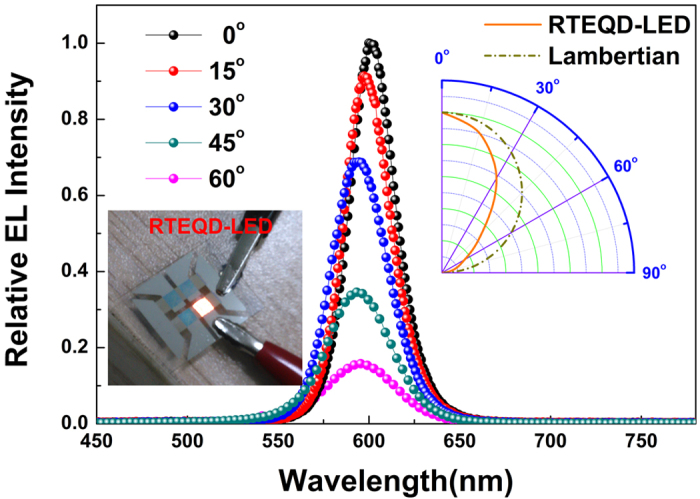
The relative EL intensity of the spectra for RTEQD-LED at different viewing angles. The inset is the photograph of the RTEQD-LED.

**Table 1:** EL performances of green and blue QD-LEDs.

Color	Type	λ_max_ (nm)	FWHM (nm)	CE_max_ (cd/A)	L_max_(cd/m^2^)	CIE
Green	BE	527	39	2.86	2152	(0.247, 0.623)
	TE	528	26	7.52	5463	(0.172, 0.757)
Blue*	BE	471	69	0.20	210	(0.167, 0.252)
	TE	468	60	0.26	951	(0.148, 0.140)

^*^Blue QD-LEDs are based on ZnSe:Cu/ZnS.
